# Post-translational Acetylation Control of Cardiac Energy Metabolism

**DOI:** 10.3389/fcvm.2021.723996

**Published:** 2021-08-02

**Authors:** Ezra B. Ketema, Gary D. Lopaschuk

**Affiliations:** Department of Pediatrics, Cardiovascular Research Centre, University of Alberta, Edmonton, AB, Canada

**Keywords:** mitochondria, fatty acid oxidation, succinylation, sirtuins, lysine acetylation, glucose oxidation

## Abstract

Perturbations in myocardial energy substrate metabolism are key contributors to the pathogenesis of heart diseases. However, the underlying causes of these metabolic alterations remain poorly understood. Recently, post-translational acetylation-mediated modification of metabolic enzymes has emerged as one of the important regulatory mechanisms for these metabolic changes. Nevertheless, despite the growing reports of a large number of acetylated cardiac mitochondrial proteins involved in energy metabolism, the functional consequences of these acetylation changes and how they correlate to metabolic alterations and myocardial dysfunction are not clearly defined. This review summarizes the evidence for a role of cardiac mitochondrial protein acetylation in altering the function of major metabolic enzymes and myocardial energy metabolism in various cardiovascular disease conditions.

## Introduction

Following the discovery of histone acetylation and its regulatory effect on RNA synthesis by Allfrey et al. (1), it has been established that alterations in the level of histone acetylation can modulate gene expressions via chromatin remodeling and epigenetic modifications (2, 3). Indeed, dysregulation of histone acetylation level has been strongly linked with the development and progression of cancer and other human diseases (4, 5). This was further reinforced by the identification of histone acetyltransferases (HAT) and histone deacetylases (HDACs), enzymes that mediate the addition or removal of an acetyl group to and from a lysine residue of histone proteins (6, 7), and the development of several HDAC inhibitors to treat cancer and heart diseases (8–10).

**Figure 1:**
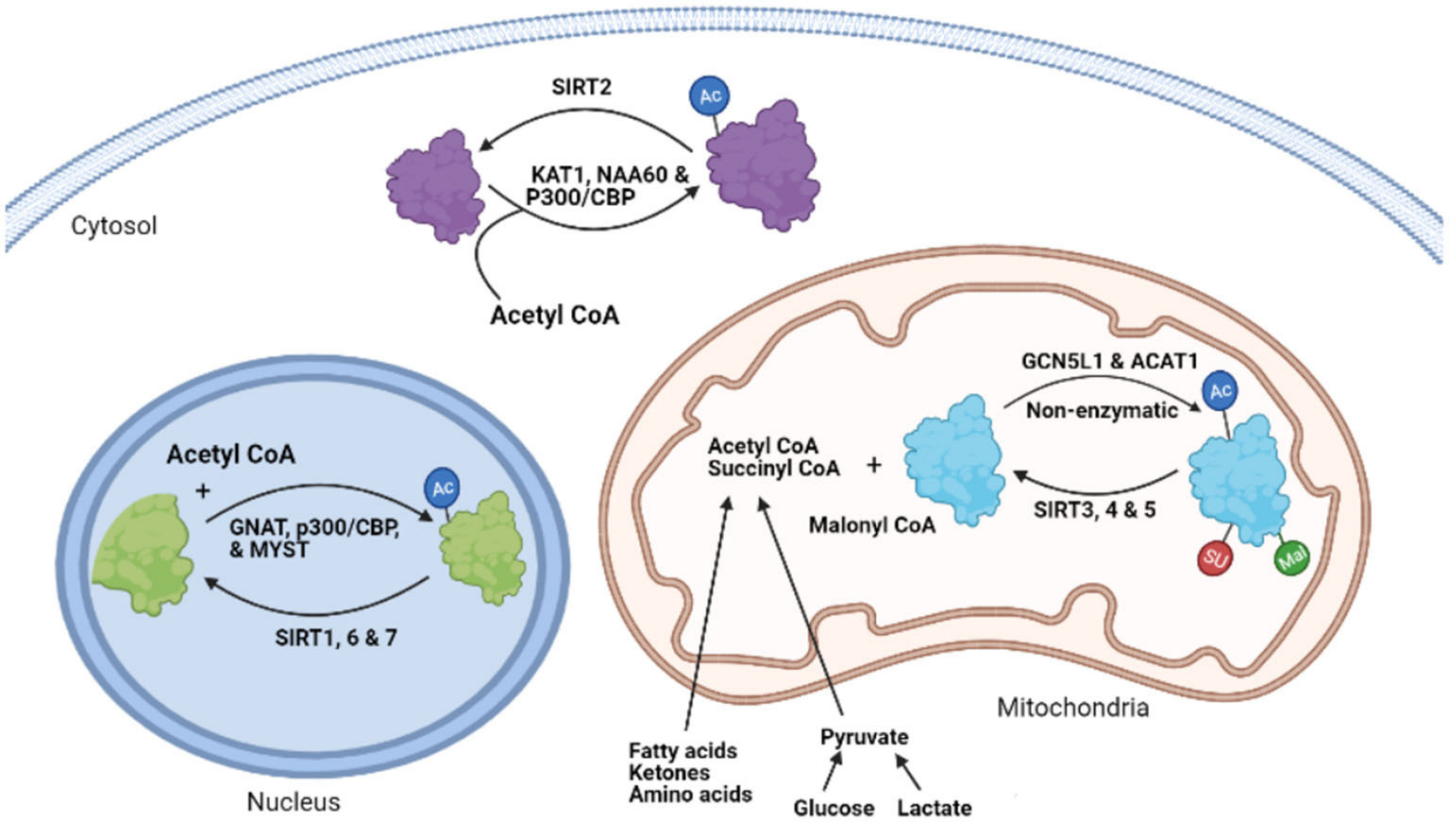
The process of protein lysine acetylation/acylation and deacetylation/deacylation. KAT1, lysine acetyltransferase; NAA60, N-α-acetyltransferase 60; CBP, CREB-binding protein; SIRT, sirtuin, GNAT, GCN5-related N-acetyltransferases; MYST, MYST family acetyltransferase; GCN5L1, General control of amino acid synthesis 5 (GCN5) like-1; ACAT1, acetyl-CoA acetyltransferase; AC, lysine Acetylation; SU, succinylation; MU, malonylation.

In addition to nuclear histone acetylation, the potential for non-histone protein acetylation of non-nuclear proteins has also recently generated considerable interest. The first acetylation of cytoplasmic proteins was described in microtubules (α-tubulin) in 1987 by Piperno et al. (11). The involvement of acetylation of non-nuclear proteins was further confirmed by the isolation of other acetylated proteins in both the cytosol and mitochondria, as well as the presence of deacetylase enzymes, such as SIRT2 and SIRT3 outside the nucleus (12–15). Since then, a number of acetylases and deacetylases have been identified outside the nucleus (Figure 1) (16). However, it is the advancements in protein acetylome quantitative methods, and the identification of several thousands of cytosolic and mitochondrial acetylated proteins using these techniques, that have recently helped advance our understanding of non-histone acetylation dynamics and its biological implications.

Kim et al. reported the first large acetyl proteomic data profile by identifying 388 acetylation sites in 195 proteins in HeLa cells and mouse liver using immunoprecipitation of lysine-acetylated peptides and mass spectrometry analysis (17). Of these, 133 proteins with 277 lysine acetylation sites were from mitochondria, showing for the first time the abundance of the acetylation process in mitochondria. Subsequently, several hundred acetylated proteins were also identified in other studies using either nutritional or acetylase/deacetylase enzymatic manipulations (18, 19). For example, in response to caloric restriction in mice, Schwer et al. reported acetylation of several proteins involved in metabolic pathways in the liver (19). Another study by Zhao et al. revealed the prevalence of acetylation of several metabolic enzymes and its possible regulatory role using both HDAC and non-histone acetylation inhibitors (20). Similarly, Lombard et al. demonstrated extensive mitochondrial protein acetylation in SIRT3 knockout (KO) mice (21), a finding confirmed by Hirschey et al. (22). Strikingly, these studies described the exceptional susceptibility of mitochondrial proteins to acetylation modification in response to various stressors, with up to 60% of mitochondrial proteins reported to being acetylated (23). Of importance, the majority of these acetylated proteins were enzymes catalyzing energy metabolic processes (19–21, 24). Similar to acetylation modifications, several studies have demonstrated increased succinylation and malonylation of a large number of cytosolic and mitochondrial proteins in response to SIRT5 deletion (25–27). For instance, in liver mitochondria isolated from SIRT5 KO mice, Rardin et al. identified 1190 succinylation sites, of which 386 sites on 140 proteins were seen in enzymes involved in energy metabolism, including fatty acid β-oxidation and ketogenesis (26). Despite these studies, that have revealed widespread acyl modifications on proteins in most of the metabolic pathways, its biological significance and regulation are still poorly understood. Understanding these post-translational modifications is particularly important in heart failure, where significant alterations in mitochondrial metabolism are seen, and disturbances in the metabolites used as a substrate for these post-translational modifications occur.

Impairments in myocardial energy substrate metabolism are a major contributor to the pathogenesis of heart failure (28–30). However, the underlying causes of these metabolic perturbations remain unclear. In the failing heart, changes in the transcription of genes encoding metabolic enzymes, particularly fatty acid metabolic enzymes, have been suggested as one of the mechanisms for altered energy substrate metabolism (31–34). Nevertheless, transcriptional regulation alone is not sufficient to explain all the metabolite and enzyme activity changes observed in the failing heart (35, 36). Several post-transcriptional and post-translational processes have been shown to modulate the original transcription products, and thus can significantly alter energy metabolism in the failing heart. Post-translational acetylation-mediated modification of metabolic proteins is thought to have a regulatory role in these energy metabolic changes (37–39).

In addition to acetylation, many other post-translational modifications including phosphorylation, malonylation, succinylation, glutarylation, ubiquitination, SUMOylation, O-GlcNAcylation, N-glycosylation, methylation, citrullination, and S-nitrosylation play important roles in cardiac disease pathogenesis, including metabolic perturbations (40–42). However, the focus of this review will be on the role of acetylation (and some acylation) modifications of energy metabolic enzymes and their contributions to altering cardiac energy metabolism. Although the relevance of protein acetylation changes have also been reported in other pathological processes, including inflammation, oxidative stress, and apoptosis, this review will mainly discuss the connections between acetylation imbalances and cardiac energy metabolic changes.

## The Process of Protein Acetylation

Lysine acetylation of proteins occurs through the covalent attachment of an acetyl group to the lysine residues of proteins. This acylation modification causes important changes to the protein at its lysine residue, which includes altering its charge status and adding an extra structural moiety (43, 44). These changes impact the proteins' native structure, its interactions with other proteins or regulatory molecules, its stability, and its function (45). Similar to acetylation, lysine succinylation, and malonylation have also emerged as functionally important acyl group modifications. These acyl modifications occur by the addition of malonyl and succinyl groups to the same or different lysine residues modified by acetylation (25, 26, 46). As discussed in the following section, cellular protein acetylation dynamics are regulated by various factors including pathological stressors, substrate availability, and the balances between acylation and deacylation enzymes (47).

While several acyltransferases have been characterized and shown to catalyze histone and other nuclear protein acetylation processes (48, 49), the involvement of these acyltransferases in the transfer of acetyl (acyl) group during cytosolic and mitochondrial protein acetylation (acylation) modifications remains to be clearly defined. A few studies suggest that some of the nuclear acetyltransferases, such as p300/CBP, may shuttle between the nucleus and cytoplasm and participate in the acetylation of cytosolic proteins (44, 50, 51). Type B lysine acetyltransferases (KATs), which include KAT1 and NAA60, are also cytoplasmic enzymes (48). The GNAT family, ATAT1 and general control of amino acid synthesis 5-like 1 (GCN5L1) acetyltransferase, also contribute to mitochondrial protein acetylation changes (Figure 1) (52, 53). However, it has also been suggested that mitochondrial protein acetylation can occur through non-enzymatic modifications (54, 55). Although widespread protein malonylation and succinylation have been described in the mitochondria (25, 56), no specific succinyltransferases or malonytransferases have been identified to date. As a result, some researchers have proposed that non-enzymatic mechanisms may be responsible for such acyl modifications (55), while others suggest that some nuclear acetyltransferases, such as histone acetyltransferase 1 (HAT1), may be involved in nuclear protein lysine succinylation (57).

Deacetylation of cytoplasmic and mitochondrial proteins mainly involves the actions of sirtuin enzymes. Sirtuins are class III NAD^+^-dependent protein deacetylases, which are considered as orthologs of silent information regulator 2 (SIR2) in yeast (58, 59). SIR2 regulates the transcription of silencing of mating-type loci, telomeres, and ribosomal DNA, thereby prolonging the yeast's lifespan (60, 61). Sirtuins can also regulate mammalian lifespan (62–64). This effect of sirtuins has led to the suggestion that sirtuins are mediators of the favorable effects of calorie restriction on health and aging, including metabolic reprogramming and stress tolerance. In support of this, caloric restriction is also shown to increase the expression of sirtuins (21, 22, 65, 66).

There are seven mammalian sirtuin proteins (SIRT1–SIRT7) with variation in their tissue specificity, subcellular localization, enzymatic activity, and targets (16). SIRT1, 6, and 7 are mainly localized in the nucleus (16, 67, 68), while SIRT2 is predominantly localized in the cytoplasm (12). However, SIRT1 and SIRT2 can shuttle between the nucleus and the cytoplasm and acetylate proteins in both compartments (67, 69). SIRT1 can regulate the acetylation state of diverse cellular proteins in the nucleus (70). In contrast, SIRT 3, 4, and 5 are mainly localized in the mitochondria (13, 16, 71), although some studies have reported cytosolic localization of SIRT5 (25). In terms of their enzymatic activity, SIRT 1–3 possess potent deacetylase activity, regulating protein acetylation status in the respective organelles (21, 23, 72). The other sirtuins, SIRT 4–7, have weak or no detectable deacetylase activity or either have very protein specific deacetylation activity (73) or mediate other deacylation processes (21). Of importance, SIRT5 has potent lysine demalonylation and desuccinylation activity (25, 56, 74). Additionally, SIRT4 and 6 have been shown to possess ADP-ribosyltransferase activity in the mitochondria and nucleus, respectively (71, 75). Also, SIRT7 has been described to have desuccinylase activity on nuclear histones (76). Combined, deacylation by sirtuins regulates diverse processes including, metabolism, gene expression, cell survival, and several other processes in the heart (77). In addition to sirtuins, recent studies have also suggested the participation of non-sirtuin HDACs in the regulation of mitochondrial acetylation dynamics (78, 79). In support of this, HDAC1 and HDAC2 have been detected in the mitochondrial isolates from mouse hearts (79).

## Myocardial Control of Acetylation/Acylation

Lysine acylation in the heart can be driven and affected by several factors including the altered level and function of acetyltransferases (such as GCN5L1) and deacylation enzymes (sirtuins), the levels of acetyl-CoA and short-chain acyl-CoAs, the levels of NAD^+^, and the underlying disease state (39, 80, 81). However, it is not yet clear how these individual factors contribute to the degree of mitochondrial protein acetylation/acylation, and whether their contribution varies according to variable conditions. As a result, despite the increased recognition of excessive protein acetylation and acylation in various forms of heart failure, there is a need to better understand the actual mechanism that is responsible for these protein post-translational modifications (PTMs).

### Altered Acyl-CoA Levels

Short-chain acyl-CoA species such as acetyl-CoA, malonyl-CoA, and succinyl-CoA are important metabolite intermediates generated during catabolism of various energy fuels. They are also donors of acetyl, malonyl, and succinyl groups for protein lysine acetylation, malonylation, and succinylation, respectively. Thus, the levels and distribution of these short acyl-CoA species can significantly affect cellular PTMs patterns.

Previous studies have suggested that increased acetylation largely arises from the non-enzymatic reaction of high levels of acetyl-CoA generated during a high-fat diet (HFD), obesity, and diabetes (82–86). Myocardial fatty acid ß-oxidation increases with a HFD, diabetes, and obesity, leading to an increase in acetyl-CoA generation (84, 87, 88). Compromised mitochondrial tricarboxylic acid (TCA) cycle activity, such as during ischemia and severe heart failure, can also increase mitochondrial acetyl-CoA levels. The mitochondrial acetyl-CoA production in these conditions may exceed the oxidative capacity of the TCA cycle and therefore increase the mitochondrial acetyl-CoA pool size. As acetyl-CoA is a substrate for acetylation, this excess acetyl-CoA has the potential to drive acetylation of mitochondrial proteins. In agreement with this, Pougovkina et al., using radioactively labeled palmitate, showed that acetyl-CoA generated by fatty acid ß-oxidation in cultured liver cells is sufficient to drive global protein hyperacetylation (89). Similarly, in a recent study, Deng et al. observed a high incorporation of fatty acid-derived ^13^C isotope onto acetylated peptides in failing mouse hearts. The authors also demonstrated a significant elevation in the levels of protein acetylation in H9c2 cells when incubated with palmitate, suggesting an association between fatty acid ß-oxidation and protein hyperacetylation (90). Wagner and Payne also demonstrated that widespread protein acetylation in the mitochondria may be facilitated by alkaline pH and high concentrations of reactive acyl-CoAs independent of any enzymatic action (55). Although these studies suggest that elevations in acetyl-CoA levels during increased fatty acid utilization enhances protein acetylation events, it has not yet been demonstrated whether an increased acetyl-CoA production from other fuels also contributes to protein acetylation modification in the mitochondria.

Unlike acetyl-CoA, the association between malonyl-CoA and succinyl-CoA levels and corresponding changes in lysine acylation in the heart has not been examined. However, in contrast to acetyl-CoA levels, malonyl-CoA levels are reduced under conditions of increased fatty acid ß-oxidation as a result of increased malonyl-CoA decarboxylase (MCD) enzymatic activity, the enzyme that degrades malonyl-CoA (91, 92). Others have also suggested that malonyl-CoA levels are unchanged during obesity or with a HFD (93, 94). High levels of fatty acids seen in these conditions also increase myocardial MCD expression, contributing to a decrease in malonyl-CoA levels (95). In contrast, increased malonyl-CoA levels in MCD deficient human fibroblast cells resulted in a two-fold increase in the levels malonylation, suggesting that malonyl-CoA levels may impact malonylation status (96). Although succinyl-CoA is one of the most abundant acyl-CoAs in the heart (46), it is not known if succinyl-CoA levels alter succinylation status in the heart. It is known that protein lysine succinylation is increased in mice hearts lacking SIRT5 (46), and that many of these proteins participate in metabolic pathways that include oxidative phosphorylation, fatty acid ß-oxidation, ketogenesis, branched-chain amino acid (BCAAs) catabolism, and the TCA cycle (97).

### Altered Expression of Acyltransferases

In contrast to the well-characterized role of multiple acetyltransferases for histone or nuclear protein acetylation, less is known regarding the role of acetyltransferases in cytosolic and mitochondrial lysine acetylation. Enzymatic acetylation of mitochondrial or cytosolic proteins may involve the GNAT family of acetyltransferases, including acetyl-CoA acetyltransferase (ACAT1) (98) and GCN5L1 (52, 53). Studies by Thapa et al. demonstrated a correlation between an excess nutrient (i.e., a HFD), upregulation of GCN5L1 expression, and increased mitochondrial lysine acetylation (53), although we observed no changes in GCN5L1 expression under similar experimental conditions (39). Reduced mitochondrial protein acetylation in GCN5L1 cardiac-specific KO mice subjected to a HFD has also been reported (99). We have also shown an increased expression of GCN5L1 in association with increased lysine acetylation in the newborn heart (100).

As discussed, protein lysine malonylation and succinylation modifications are highly prevalent in enzymes of mitochondrial metabolism and the TCA cycle (25, 56, 74). However, it is still unknown whether these processes are catalyzed by succinyl or malonyl transferases or whether they occur passively. Therefore, it remains unclear how these protein acylation modifications are regulated during pathological conditions.

### Altered Expression of Sirtuins

SIRT3 is a major mitochondrial deacetylase. Studies have shown an association between SIRT3 deletion and mitochondrial protein hyperacetylation, supporting its critical deacetylating role (21, 22). Many key enzymes in fatty acid and carbohydrate metabolism are substrates for SIRT3 deacetylation (72, 101, 102). Downregulation of SIRT3 occurs in response to stressors such as a HFD or various heart diseases (81, 103). Decreased SIRT3 has been also implicated in various cardiac pathologies in association with hyperacetylation of mitochondrial proteins (54, 104). For instance, a change in the expression of SIRT3 isoforms (long and short forms) is seen in mice hearts subjected to different hypertrophic stimuli (105). However, there is a lack of understanding as to how SIRT3 gene expression is affected by altered metabolic (nutrient) state or heart failure. Moreover, most previous studies have focused on the expression levels of SIRT3, as opposed to actual SIRT3 enzymatic activity.

Cardiac SIRT1, a deacetylase enzyme in the nucleus and cytosol, is also downregulated in advanced heart failure (106). Similar findings in other heart failure studies have also been observed, which demonstrated an association between decreased SIRT1 expression and increased oxidative stress (107). Paradoxically, other researchers have shown a correlation between constitutive overexpression of SIRT1 and impaired cardiac function, as well as disturbed cardiac energy metabolism in response to acute pressure overload (108). SIRT1 protein is also negatively regulated by HFD, which induces its cleavage by the inflammation-activated caspase-1 in adipose tissue (109).

Some studies have indicated a high level of SIRT5 expression in normal hearts (16). However, the pattern of changes in SIRT5 levels under stress conditions is not well-characterized. A previous study on mouse primary hepatocytes have suggested upregulation of SIRT5 by peroxisome proliferator-activated receptor coactivator-1α (PGC-1α), and downregulation by AMP-activated protein kinase (AMPK) (110). Unlike SIRT1 and 3, the absence of SIRT5 does not affect the development of HFD-induced metabolic abnormalities and insulin resistance (111).

HDACs are known to modulate histone acetylation status and thus affect its interaction with DNA, which results in chromatin remodeling and transcriptional changes (2). However, recent studies have also implicated a role for HDAC in modifying the mitochondrial acetylome directly in a non-transcriptional manner using various HDAC inhibitors (78, 79, 112). Both hyperacetylation and hypoacetylation of mitochondrial proteins was observed in response to a pan-HDAC inhibitor in a feline model of heart failure (78). However, the effects of these acetylation modifications was not investigated in this study. Moreover, though a positive association has been made between HDAC inhibition and improved cardiac function in relation to acetylation changes, it has not yet been explored how HDAC inhibition affects sirtuin functions or whether the patterns of acetylation regulated by HDAC inhibition is different from those regulated by mitochondrial sirtuins.

### Altered NAD^+^ Levels

NAD^+^ is an important cofactor for sirtuins, and as such fluctuation in NAD^+^ levels may be one of the contributing factors for altered protein acetylation levels (58). Through NAD^+^, sirtuin activity is directly linked to the energy status of the cell. NAD^+^ is synthesized from different biosynthetic precursors. In the salvage pathway, the major NAD^+^ generating pathway, nicotinamide riboside (NR) and nicotinamide (NAM) are converted into nicotinamide mononucleotide (NMN) by nicotinamide riboside kinase (NRK) and nicotinamide phosphoribosyltransferase (NAMPT) enzymes, respectively. Nicotinamide mononucleotide adenyltransferases (NMNAT) then converts NMN to NAD^+^. In the *de novo* pathway, NAD^+^ is generated from the amino acid tryptophan, which is ultimately converted into the biosynthetic intermediate, nicotinic acid mononucleotide (NaMN) through multiple enzymatic steps. Nicotinic acid mononucleotide is then converted to nicotinic acid dinucleotide (NaAD^+^) by NMN/NaMN adenylyltransferases (NMNATs) and then converted to NAD^+^ by NAD^+^ synthetase through deamination (113). Intracellular NAD^+^ levels can also be altered by rates of glycolytic and mitochondrial metabolic pathways using NAD^+^ to produce NADH, rates of mitochondrial electron transport chain activity that produce NAD^+^ from NADH, and by enzymes that consume or catabolize NAD^+^, such as the poly ADP-ribosyltransferases (PARPs) and the NAD^+^ cyclases (CD38) (113, 114).

Alterations in the NAD^+^ biosynthetic or degradation pathways may directly affect the activity of sirtuins and thus protein acetylation status (115, 116). Kinetics studies on sirtuins and NAD^+^ metabolites, have demonstrated the sensitivity of sirtuins to changes in nicotinamide and NAD^+^ levels, which inhibits and activates its enzymatic activity, respectively (58, 117, 118). While both the NADH and NAD^+^/NADH ratio have been previously suggested to impact lysine acetylation status (80, 119, 120), recent studies indicated that alterations in NADH have insignificant effect on sirtuin regulation (117, 121). Accordingly, NADH has a very poor binding affinity to sirtuins (117), and sirtuins are insensitive to NADH inhibition at the concentration of NADH found in the cell (121). As a result, changes in NAD^+^, as opposed to commonly reported changes in the NAD^+^/NADH ratio, should be used for assessing NAD^+^ regulation of sirtuin activity (122). Both NADH & NAD^+^, as well as NAD^+^/NADH ratio, also significantly varies across cellular compartments and in response to various disease or metabolic states, making it difficult to interpret the implication of NAD^+^/NADH ratio in controlling sirtuin activity (122). In addition, the NAD^+^/NADH ratio alone does not also provide specific information on the direction of changes to the individual nucleotides. Thus, measurement of free NAD^+^ levels is most relevant when it comes to the regulation of sirtuins and perturbations in protein acetylation.

Previous studies have demonstrated changes in the activity of sirtuins and protein acetylation levels by modulating both NAD^+^ synthetic and catabolic pathways (41, 115, 123). Accordingly, Lee et al. observed a significant decrease in the mitochondrial proteins acetylome in response to NAMPT overexpression or NAD^+^ supplementation (80). Similarly, other studies have also shown increased NAD^+^ levels accompanied by enhanced SIRT1 and SIRT3 activities in responses to NR supplementation, which was accompanied by an increase in oxidative metabolism and protection against HFD-induced metabolic abnormalities (123). Supporting this, producing a CD38 deficiency (which is a NAD^+^ degrading enzyme) protects the heart from HFD-induced oxidative stress by increasing NAD^+^ availability and activating SIRT3 mediated protein deacetylation (115). NAD^+^ depletion occurs in many cardiac pathologies, such as during ischemia-reperfusion (I/R) injury, and several therapeutic strategies to increase NAD^+^ levels have been proposed (124, 125). However, the mechanisms which mediate the favorable effects of increasing NAD^+^ levels are not completely understood, although emerging data suggests activation of sirtuins and decreasing protein acylation modifications as key effectors (126–129).

## Acetylation/Acylation of Energy Metabolic Enzymes and Myocardial Metabolic Alterations

Alterations in myocardial energy metabolism, both in terms of changes in energy substrate preference, and decreased mitochondrial oxidative metabolism and ATP production, are key contributors to heart failure development (28, 130–133). Various injury or stress signals, including ischemia, hypertrophy or neurohormonal changes, are thought to mediate these metabolic derangements (130). While these disturbances result in an unbalanced use of glucose and fatty acids and a decreased contractile efficiency during heart failure, it remains controversial whether the shifts occur toward increased glucose use or increased fatty acid use, and whether these shifts are adaptive or pathological (30, 33, 134–139). Our limited understanding of the underlying mechanisms regulating these metabolic changes is a major challenge to better characterizing these alterations for potential therapeutic interventions.

Changes in metabolic gene expressions, predominantly down-regulation of fatty acid transporting and metabolizing proteins, have been described as one of the contributors to the metabolic changes seen in heart failure (31–34, 139). Recently, apart from transcription regulation, several post-transcriptional and post-translational processes have been shown to modulate transcriptional and protein products (41, 42). There is also an inconsistency in these transcriptional changes, within both the metabolic pathways and across species (31). For instance, while downregulation of fatty acid metabolic enzymes expression is observed in various animal models of heart failure, no significant alterations are observed in genes regulating glucose metabolism, or changes are largely inconsistent, in human heart failure samples (31, 35). Even within the fatty acid metabolic genes, transcriptional downregulation has been observed only in a few of them compared to the widespread post-translational modification seen in most of these enzymes (34, 35). In support to this, Sack et al. observed, a mismatch between mRNA levels and activities of some of the fatty acid metabolic enzymes in the failing heart (139), suggesting a role for post-transcriptional and post-translational changes.

**Figure 2:**
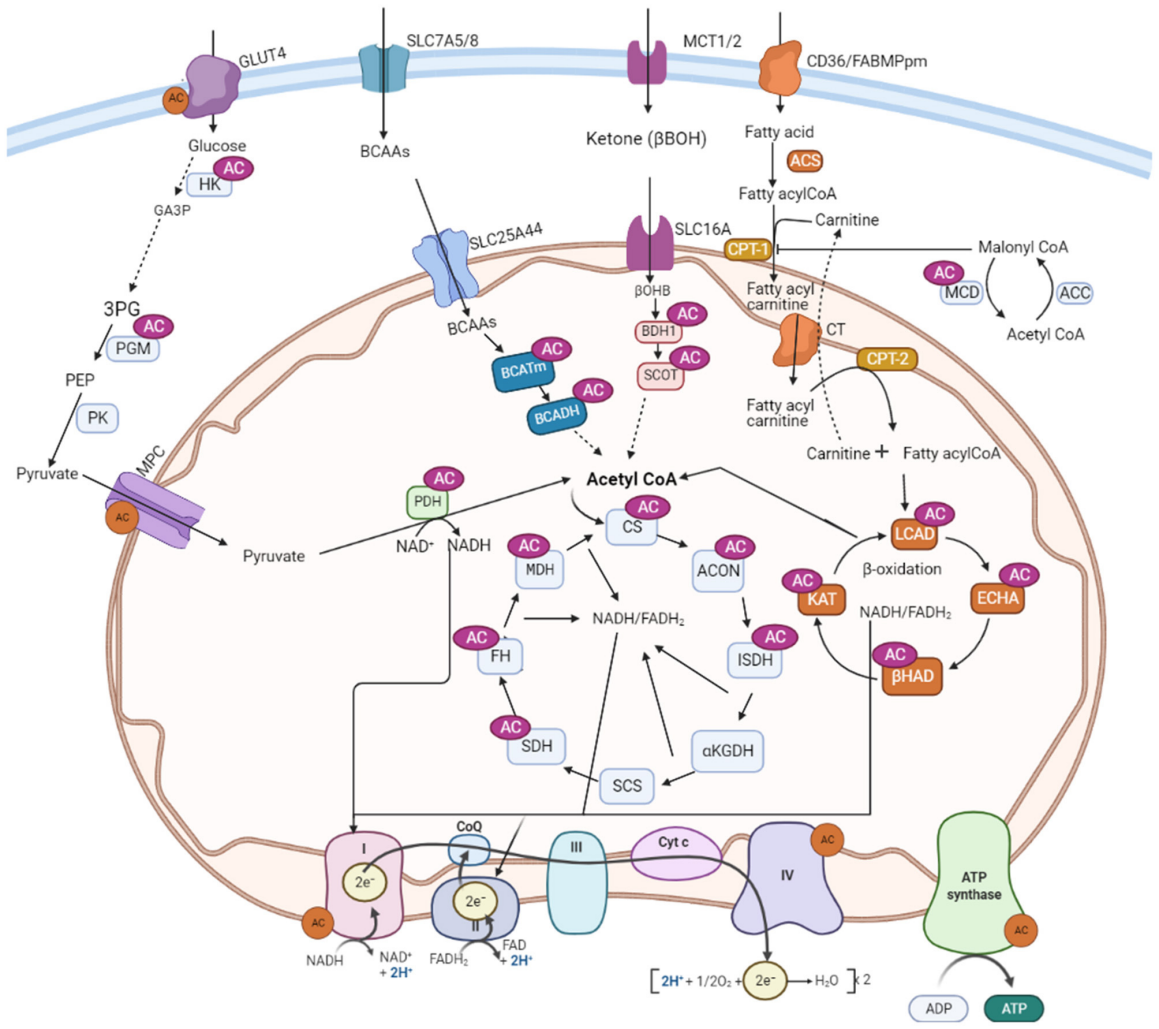
Metabolic proteins subjected to acetylation control in the heart. AC, lysine acetylation; GLUT4, glucose transporter isoform 4; SLC7A5/8, solute carrier family-7; SLC25A44, solute carrier family-25; SLC16, solute carrier family-16; MCT, monocarboxylate transporter 1; CD36, cluster of differentiation 36; FABPpm, plasma membrane fatty acid-binding protein; MCD, malonyl CoA decarboxylase; ACC, acetyl CoA carboxylase; PDH, pyruvate dehydrogenase; LCAD, long-chain acyl CoA dehydrogenase; β-HAD, β-hydroxyacyl CoA dehydrogenase; KAT, 3-ketoacyl-coa thiolase; ECH, enoyl-CoA hydratase; FAS, fatty acyl CoA synthase; CPT-1, carnitine palmitoyltransferase 1; CPT-2, carnitine palmitoyltransferase-2; CT, carnitine acyl translocase; BCAA, branched-chain amino acids; BCATm, mitochondrial branched-chain aminotransferase; BCADH, branched-chain amino acid dehydrogenase; β-OHB, β-hydroxybutyrate; BDH-1, 3-hydroxybutyrate dehydrogenase 1; SCOT, 3-ketoacid CoA transferase; CS, citrate synthase; ISDH, iso-citrate dehydrogenase; α-KGDH, alpha-ketoglutarate dehydrogenase; SCS, succinate CoA synthetase; MDH, malate dehydrogenase; SDH, succinate dehydrogenase; FH, fumarate hydratase; OAA, oxaloacetate; MCD, malonyl CoA decarboxylase; ACON, aconitase; HK, hexose kinase; GA3P, glyceraldehyde 3-phosphate; 3PG: 3-phosphoglycerate; PGM, Phosphoglycerate mutase; PK, pyruvate kinase; CoQ, coenzyme Q; Cytc: cytochrome C; FAD/FADH_2_, flavin adenine dinucleotide; NAD/NADH2, nicotinamide adenine dinucleotide.

Growing evidence suggests that post-translational acetylation modification may play a significant role in altering myocardial metabolism during heart failure by modifying the function and structure of major metabolic proteins (39, 104, 140). Hyperacylation of key metabolic enzymes involved in fatty acid and glucose metabolism has been shown in heart failure as well as response to excess nutrition or sirtuin deletions (81, 83, 97, 141, 142). However, despite the modification of many of these enzymes by acetylation or other acylations, the actual impact of these PTMs on individual pathways and enzymes remains poorly understood. In this section, we summarize recent evidence on the impact of hyperacetylation on selected metabolic enzyme activity in the heart as well as in other organs or cells (Figure 2).

### Fatty Acid ß-Oxidation

The enzymes catalyzing the cyclic reactions of fatty acid β-oxidation (which converts fatty acid carbons into acetyl-CoA moeities) includes long-chain acyl CoA dehydrogenase (LCAD), enoyl-CoA hydratase, L-3-hydroxy acyl-CoA dehydrogenase (β-HAD), and 3-ketoacyl-CoA thiolase (KAT) (143, 144). Acetylation of these enzymes has been widely reported in various studies (Table 1) (20, 22, 83). The functional consequences of acetylation are relatively well-studied for LCAD and β-HAD. However, most of these studies were conducted in the liver and skeletal muscle, and only a few studies examined the direct impact of acetylation on fatty acid metabolism in the heart.

**Table 1:** Effect of lysine acetylation on major metabolic enzymes in the heart.

**Metabolic pathway**	**Enzymes**	**Effect on enzyme activity**	**References**
Fatty acid oxidation	LCAD	↑, ↓	(53, 83, 145)
	β-HAD	↑	(53, 83)
	MCAD	↑	(146)
	MCD	↑	(73)
Glucose oxidation	PDH	↓	(53, 83, 147)
	MPC	↓	(148)
Glycolysis	HK	↓	(100)
	PGM	↓	(100)
	GLUT4	↓	(149)
Insulin signaling	Akt	↓	(147, 150)
TCA cycle	ICDH	↓,⇔	(151)
	SDH	↓	(81)
	ACON	↑	(152)
ETC	Complex I	↓	(24, 153)
	Complex III	↓	(24)
	Complex V	↓	(24, 153)

*GLUT4, glucose transporter isoform 4; PDH, pyruvate dehydrogenase; LCAD, long-chain acyl CoA dehydrogenase; β-HAD, β-hydroxyacyl CoA dehydrogenase; ICDH, iso-citrate dehydrogenase; MCAD, medium-chain acyl CoA-dehydrogenase; MCD, malonyl CoA decarboxylase; MPC, mitochondrial pyruvate carrier; SDH, succinate dehydrogenase; ETC, electron transport chain; Akt, protein kinase B; PGM, phosphoglucomutase; ACON, aconitase; HK, hexokinase; TCA, tricarboxylic acid; ↑, increased activity; ↓, decreased activity; ⇔, no change in the activity of the enzyme*.

The exact effect of acetylation on fatty acid metabolizing enzymes' activity remains controversial, and there are two opposing views. Using both HFD and SIRT3 KO mice, we demonstrated a positive correlation between increased acetylation of myocardial LCAD and β-HAD and their enzymatic activities as well as increased fatty acid ß-oxidation rates in the heart (83). A similar relationship between acetylation and increased fatty acid ß-oxidation was also seen in newborn rabbits and human hearts. In the early newborn period, a dramatic maturational change in myocardial energy substrate metabolism occurs, accompanied by an increase in fatty acid ß-oxidation. In association with this, we have shown increased acetylation of LCAD and β-HAD, accompanied by an increase in their activities, during the maturation of fatty oxidation in neonatal rabbit hearts (100). The increased acetylation of these enzymes is accompanied by an up-regulation of the acetyltransferase, GCN5L1. In a separate study, we also found a decreased LCAD and βHAD activities and a decrease in fatty acid ß-oxidation rates in hypertrophied newborn human and rabbit hearts in association with decreased acetylation status of these enzymes (154). Similarly, Thapa et al. revealed a positive association between increased acetylation and activities of several cardiac fatty acid ß-oxidation enzymes, including LCAD, β-HAD, and short-chain acyl-CoA dehydrogenase in chronic HFD mice, along with GCN5L1 upregulation (53). Furthermore, decreasing acetylation by GCN5L1 knockdown leads to diminished fatty acid ß-oxidation in cultured H9C2 cells, supporting the idea that lysine acetylation promotes fatty acid ß-oxidation in the heart (53).

Additional evidence for a positive correlation between increased acetylation and increased activities of fatty acid ß-oxidation enzymes have been reported from studies in diabetic animals. In streptozotocin-induced type 2 diabetic rat hearts, Vazquez et al. found a significant increase in mitochondrial lysine acetylation compared to the controls (143). Increased activities of medium- and long-chain acyl-CoA dehydrogenases (MCAD, LCAD) and fatty acid ß-oxidation rates were observed in the hearts of diabetic animals. Analysis of substrate preference in these hearts also revealed an increase in state 3 respiration using palmitoylcarnitine as a substrate (146). Similarly, in type 1 diabetic mice, a 2.5-fold increase in total acetylation levels compared to age-matched controls was observed in the heart. In this study, the maximal rate of respiration remained unchanged only when palmitoylcarnitine or a fatty acid-based substrate was used (85). Furthermore, data from the two most recent studies also indicated that fatty acid utilization in the heart is either unaffected or proceed in harmony with increased acetylation state (90, 155).

Activation of fatty acid ß-oxidation by acetylation is also reported in other tissues/cells. A direct relationship between hyperacetylation and activity of enoyl-coenzyme A hydratase/3-hydroxy acyl-coenzyme A was demonstrated in the presence of high fat and deacetylase inhibitors in cultured Chang human liver cells (20). Other investigators have also found a link between HDAC3 mediated deacetylation of 3-hydroxy acyl-coenzyme A and its decreased activity in macrophages, while HDAC3 depletion reversed this effect (112). Moreover, in SIRT3 KO mice and SIRT3 deficient skeletal muscle cells, Jing et al. showed an increase in palmitate oxidation rates in the presence of excessive acetylation (155). In the presence of high palmitate, oxygen consumption rates are significantly higher in SIRT3 lacking myoblasts, which is lost in the presence of etomoxir, a fatty acid ß-oxidation inhibitor (156). Together, these studies demonstrate that increased acetylation of myocardial fatty acid ß-oxidation enzymes is associated with their enhanced activities. This is further supported by the fact that both myocardial fatty acid utilization and mitochondrial acetylation are enhanced during a HFD, obesity, and diabetes (157). The high fatty acid ß-oxidation rate seen in these conditions may also lead to the increased production of acetyl-CoA that can serve as a substrate for acetylation (82). Thus, it is reasonable to assume that increased acetylation of fatty acid oxidative enzymes can further trigger the enzyme activity and led to the continuation of fatty acid ß-oxidation in the heart in these circumstances.

In contrast to the scenarios discussed above, a study by Koentges et al., in isolated working hearts, found a negative correlation between hyperacetylation of LCAD and its activity along with reduced palmitate oxidation in SIRT3 deficient transverse aortic constriction (TAC) mice (158). However, these hearts were perfused with a buffer that contained an ultra-physiological high concentration of glucose (11 mM) and a lower fatty acid to albumin ratio (1.5%) where both conditions may contribute to decreased fatty acid ß-oxidation rates. Likewise, Chen et al. also reported an abnormal lipid accumulation and decreased palmitate ß-oxidation rates in TAC-induced hypertrophic hearts, with a further decline in SIRT3 KO mice hearts in association with hyperacetylation of LCAD (145). A recent study by Davidson et al. showed reduced expression of genes of fatty acid catabolism despite no overt abnormalities in mitochondrial respiration in mice deficient for cardiac carnitine acetyltransferase and SIRT3, despite the fact that the hearts exhibited extreme acetylation (159). In common, these three studies were done in mice with TAC-induced heart failure, while the last two did not assess directly the acetylation status of the enzymes. Overall, it is not clear if TAC alters the dynamics of acetylation on LCAD differently, such as through distinctive sites or multiple site modifications. However, several acyl modifications may likely coexist under these circumstances, which possibly compete with acetylation for the same lysine residue (104). However, experimental data are lacking regarding these interactions in heart failure.

Unlike the heart, studies conducted in the liver reported an inhibitory effect of acetylation on fatty acid metabolism. Hirschey et al. described a decreased activity of LCAD enzyme and reduced fatty acid ß-oxidation following hyperacetylation of these enzymes in SIRT3 KO mice. The authors further reported an accumulation of long-chain acylcarnitine species, fatty acid ß-oxidation intermediate products, and triacylglycerol in livers from SIRT3 KO mice that could suggest a decreased rate of fatty acid ß-oxidation. From the eight acetylated lysine residues detected on LCAD, lysine residue 42 was identified as a critical regulation site for acetylation (22). Furthermore, analysis of the rate of conversion of radiolabeled palmitate revealed lowered oxidizing capacity and ATP production in tissue homogenates from SIRT3–/– compared to wild-type tissue at a high substrate concentration. Reduced activities of fatty acid ß-oxidation enzymes in the liver by acetylation were also reported in other studies (160). In addition to LCAD and β-HAD, hyperacetylation of hydroxy acyl-CoA dehydrogenase, another important enzyme in fatty acid ß-oxidation, led to its decreased activity and decline in overall fatty acid oxidation rate in the mouse liver. Deletion of GCN5L1 acetylase enzyme or overexpression of SIRT3 reduced hydroxy acyl-CoA dehydrogenase acetylation and increased its activity as well as fatty acid ß-oxidation in the liver (161, 162). However, there is presently no agreement on the functionally significant acetylation sites or SIRT3 target sites in these studies. While Hirschey et al. noted lysine 42 residue on LCAD as an important regulation site (22), using chemically acetylated recombinant proteins, Bharathi et al. identified Lys-318 and Lys-322 residues as an important site for LCAD acetylation/sirt3 deacetylation (160). To date, a detailed analysis of lysine residue modification and functional acetylation/deacetylation target sites for LCAD and β-HAD is lacking in the heart. Not all lysine residues within LCAD that are acetylated are expected to impact LCAD activity in the same manner. Understanding the acetylation status of different acetylation sites, and their effect on the enzyme function in multiple tissues will help to characterize the tissues specific effects of acetylation dynamics.

Compared to acetylation, the effect of succinylation and malonylation modification on fatty acid ß-oxidation enzymes is not clear. Some studies have shown an impaired β-oxidation and accumulation of medium- and long-chain acylcarnitines in the liver and muscles of SIRT5 KO mice (26). Most of the acyl-CoA dehydrogenase enzymes, including very long-chain acyl-CoA dehydrogenase (VLCAD), LCAD, and MCAD, were found to be hypersuccinylated, suggesting a suppressive effect of excessive succinylation on fatty acid oxidizing enzymes (26). In contrast, cardiac ECH is desuccinylated and activated by SIRT5 (46, 97). But the effects of similar modifications on other enzymes in this pathway have yet to be determined.

Overall, from all these data it is possible to suggest that the effect of lysine acetylation on fatty acid ß-oxidative enzymes may not be similar between different tissues, at least between the liver and heart. Reasonably, these differences may account for the specialization of these tissues in regulating fatty acid ß-oxidation differently in line with their physiological functions. The liver has a high capacity for both synthesizing and oxidizing fatty acids and the two processes are regulated reciprocally during a fed or fasting state as well in disease conditions such as in obesity or diabetes. On the contrary, the heart continually uses fatty acids as a source of energy, which accounts for up 60–90% of the total energy requirements for its normal contractile function irrespective of the fed state or disease conditions. While acetylation may serve as a feedback regulation in the liver as suggested by Bharathi et al. (160), the same mechanism would potentially compromise the heart's ability to produce energy if fatty acid ß-oxidation enzymes were down-regulated by acetylation induced by excess fatty acid ß-oxidation. Thus, future studies are needed to determine the differences in acetylome between the two tissues, and if acetylation-mediated regulation of fatty acid ß-oxidation is tissue- or site-specific.

### Glucose Metabolism

#### Glucose Oxidation

In contrast to fatty acid metabolism, the effects of acetylation on glucose metabolism have received less attention, especially in the heart. However, recent studies reported the acetylation of several proteins involved in glucose transport, glycolysis, and glucose oxidation (83, 149, 156). The pyruvate dehydrogenase complex (PDH) is one of the most widely investigated glucose metabolizing enzymes in relation to acetylation. It is a key enzyme that catalyzes the irreversible and rate-limiting step in glucose oxidation that links glycolysis to the TCA cycle. PDH is regulated by several mechanisms, but the change in its phosphorylation status is critical to its activity. It is inhibited by phosphorylation on the E1 subunit by a specific PDH kinase (PDK), PDK4, and is activated when dephosphorylated by PDH phosphatase (163, 164).

Modification of PDH by acetylation has been demonstrated in several studies (53, 83, 156). In HFD induced obese mice subjected to a TAC, we showed a significant increase in PDH acetylation with a decrease in glucose oxidation rates (147). Similarly, Thapa et al. reported increased acetylation of the α-subunit of PDH in the heart after HFD feeding, and its hyperacetylation was shown to inhibit its activity (53). Reduced activity of PDH in association with its increased acetylation and decreased SIRT3 level has been also demonstrated in mice with angiotensin II-induced cardiac hypertrophy (165).

In addition, increased acetylation has also been implicated in a reduced transport of pyruvate into the mitochondria. Akita type 1 diabetic mice hearts exhibit a significant hyperacetylation state, along with a 70% decrease in the rate of mitochondrial pyruvate transport that occurs without any changes in the protein level of the mitochondrial pyruvate carriers 1 and 2 (MPC1 and MPC2). Mass spectrometry analysis revealed that acetylation of lysines 19 and 26 of MPC2 were increased in Akita mice heart mitochondria, and that acetylation at these sites is associated with impaired pyruvate metabolism in the heart (148).

The impact of acetylation on PDH has also been examined in skeletal muscle by Jing et al. Deletion of SIRT3, both *in vivo* in SIRT KO mice and *in vitro* in myoblasts, lead to a significant increase in acetylation associated with decreased PDH activity along with a reduced glucose oxidation rate and accumulation of pyruvate and lactate metabolites. Six acetylation sites have been identified on the PDH E1α subunit, with lysine 336 being significantly altered by SIRT3 deletion (156). Interestingly, it was also shown that hyperacetylation of the PDH E1α at lysine 336 enhances its phosphorylation leading to suppressed PDH enzymatic activity. Additionally, Ozden et al. and Fan et al. also demonstrated the inhibitory effect of acetylation at lysine 321 on PDH activity in cancer cells, which is completely reversed by SIRT3 activation (97, 164). In the latter study, it was also shown that lysine acetylation at lysine 202 inhibits PDP1 by dissociating it from PDHA1, thus promoting its phosphorylation (97).

Hypersuccinylation of PDH accompanied by a decrease in SIRT5 expression is also associated with a decrease in PDH activity as the heart maturates in postnatal rabbits (100). Unexpectedly, Park et al. observed the suppressive effect of SIRT5 catalyzed desuccinylation on PDH in *SIRT5* KO mouse embryonic fibroblasts (MEFs), while SIRT5 deletion led to an increase in PDH activity (25). In contrast, Zhang et al. found significantly reduced malate/pyruvate-driven respiration in SIRT5-deficient HEK293 cells, as well as in homogenates prepared from SIRT5 KO livers (166). While these discrepancies need further investigation, tissue/cell-specific variation in these modifications may contribute to these differences.

#### Glycolysis

Acetylation of glycolytic enzymes has been reported in hearts as well as various other cells. In newborn rabbit hearts, we showed a significant decline in glycolysis rates in line with hyperacetylation of its enzymes, including hexokinase (HK-1) and phosphoglycerate mutase (PGM) (100). In contrast, Hallows et al. have shown a negative regulation of PGM by SIRT1 (165). Acetylated PGM displayed enhanced activity, while Sirt1-mediated deacetylation reduced its activity in human embryonic kidney (HEK293) cells (167). Glyceraldehyde-3-phosphate dehydrogenase (GAPDH) is another glycolytic enzyme subjected to acetylation modification. Similar to PGM, acetylation of GAPDH at lysine 254 increases its enzymatic activity in response to glucose in HEK293T cells (168). In contrast, GAPDH acetylation enhances its translocation from the cytoplasm to the nucleus in NIH3T3 cells, thereby inhibiting downstream glycolysis and accumulation of glycolytic intermediates (169). In another study, Xiong et al. showed an inhibitory effect of acetylation of pyruvate kinase (PK), which catalyzes the last step of glycolysis (170). In addition to acetylation, other acyl modifications can regulate glycolysis. GAPDH, PGK, and enolase are hypermalonylated in livers of *db/db* mice (142), although the effects of this hypermalonylation on the activities of these enzymes has not been investigated. Another study in hepatocytes demonstrated SIRT5 mediated demalonylation of GAPDH and increased activity, suggesting that malonylation decreases glycolytic flux (27). Similarly, desuccinylation of PK by SIRT5 increases its kinase activity (171). In contrast, Xiangyun et al. reported that desuccinylation of PKM2 by SIRT5 inhibits its activity in tumor cells (172). Unfortunately, there is a lack of data on the effect of malonylation and succinylation modifications on glycolysis in the heart.

At the transcriptional level, glycolysis is regulated by the level of hypoxia-inducible factor-1α (HIF-1α), a master transcriptional regulator of glycolytic enzymes (173). Studies by Geng et al. have shown a positive correlation between increased acetylation of HIF-1α by p300 at lysine 709 and its stability, or decreased polyubiquitination, in HEK293 cells (174). Similarly, other studies also showed an inhibition of HIF-1α by SIRT1 mediated deacetylation at lysine 674, in HT1080 and HEK293 cells (175). These results were also found in SIRT6 deficient embryonic stem cells and MEFs cells (174). Interestingly, these cells exhibit increased glucose uptake with up-regulation of glycolysis in association with increased HIF-1α activity.

The impact of acetylation on glucose uptake and its transporters has also been described. In both cultured cardiomyocytes and perfused hearts, Renguet et al. found that acetylation of GLUT 4 inhibits glucose uptake in adult cardiomyocytes, as well as in perfused hearts, by decreasing its translocation to the plasma membrane (173). Strikingly, treatment with inhibitors of acetyltransferases prevents the increase in protein acetylation and reverses the inhibition of glucose uptake and GLUT4 translocation (149). Unfortunately, the direct acetylation status of GLUT4 was not analyzed in this study. However, the inhibitory effect of acetylation on glucose uptake is supported by other studies. Using SIRT3 KO mice and hyperinsulinemic-euglycemic clamp experiments, Lantier et al. showed that increased acetylation leads to insulin resistance and reduced muscle glucose uptake that is associated with decreased hexokinase II (HKII) binding to the mitochondria in HFD-fed SIRT3 KO mice (176). This suggests a reduced HKII activity and translocation as a result of increased acetylation. Similar to the above study, unfortunately, there was no direct analysis of the acetylation status of the proteins involved in glucose uptake or glucose phosphorylation.

#### Insulin Signaling

Insulin resistance in type 2 diabetes and obesity as well as in other heart conditions contributes to a number of adverse changes in the heart that includes alterations in cardiac energy metabolism, lipotoxicity, and hypertrophy, and is associated with an increased risk of heart failure (84, 177, 178). Cardiac insulin signaling is impaired in heart failure, diabetes, and obesity (147, 178, 179). Recent studies have shown that several proteins in the insulin signaling pathway are targets for acetylation modification, which therefore may impact insulin signaling. Akt is an important component of the insulin signaling pathway. Akt activation requires binding with phosphatidylinositol 3,4,5-trisphosphate [PIP (3)], which promotes its membrane localization and phosphorylation by the upstream kinase, phosphoinositide-dependent protein kinase 1 (PDK1). Previously, we have shown a negative association between acetylation of insulin signaling mediators, such as Akt and PDK1, and their decreased activation as a result of changes in their phosphorylation status due to acetylation (147). In support of this, Sundaresan et al. showed that acetylation of Akt and PDK1 occurs in their pleckstrin homology (PH) domains, which blocks PIP (3) binding, and that this is reversed by SIRT1 deacetylation (150). SIRT2 also binds and activates Akt in insulin-responsive cells, through its interaction with the PH domain, whereas SIRT2 inhibition impairs AKT activation by insulin (180).

### Acetylation and Metabolism of Other Fuel Substrates

Ketone body and BCAA oxidation can impact cardiac energy metabolism and heart failure progression (179, 181, 182). Both pathways may also contribute to mitochondrial acetylation changes. However, only a few studies have characterized the acetylation status and its impact on enzymes involved in ketone and BCCA metabolism.

In hepatic mitochondria of SIRT3 KO mice, hydroxymethyl-glutaryl (HMG)-CoA synthase (HMGCS2), the rate-limiting enzyme in ketogenesis, is hyperacetylated and its enzymatic activity reduced, leading to a decrease in β-hydroxybutyrate synthesis. Deacetylation of HMGCS2 by SIRT3 increases its enzymatic activity and β-hydroxybutyrate levels (183). Similar to the acetylation effect, loss of SIRT5 results in hypersuccinylation of HMGCS2 and reduces its activity both *in vivo* and *in vitro* in liver mitochondria, which leads to reduced β-hydroxybutyrate levels during fasting (26).

With regard to ketone oxidation, succinyl-CoA:3-ketoacid-CoA transferase (SCOT), a key enzyme of ketone oxidation, is hyperacetylated in the brain and heart at multiple sites in SIRT3 KO mice (184). *In vitro* biochemical analysis of recombinant SCOT demonstrates that acetylation at lysine 451 residues results in decreased enzyme activity that is reversed by SIRT3 activation. Moreover, in brain homogenates from WT and SIRT3 KO mice, acetoacetate-dependent acetyl-CoA production is decreased by three-fold in SIRT3 KO mice, suggesting decreased ketone oxidation rates upon increased acetylation (180). In contrast, a decrease in ketogenesis capacity was noted in the liver of mice lacking SIRT3 (184). However, similar studies are lacking in the heart.

Similarly, enzymes in BCAA (isoleucine, leucine, and valine) catabolic pathways are among the proteins regulated by acetylation and SIRT3 in the liver. Some acetylation sites were detected in branched-chain alpha-keto acid dehydrogenase (BCKDH), a key enzyme catalyzing the breakdown of BCAAs, in SIRT3 KO mice (181, 182). As BCAA levels were raised, the authors suggested that acetylation may have an inhibitory effect on branched-chain ketoacid dehydrogenase (BCKDH) activity (23, 185). Other investigators have also suggested that acetylation of BCAA aminotransferase (BCAT) promotes its degradation in the ubiquitin-proteasome pathway, thereby decreasing BCAA catabolism in the pancreas (186). cAMP-responsive element-binding (CREB)-binding protein (CBP) and SIRT4 were identified as the acetyltransferase and deacetylase for BCAT at lysine 44 (K44), respectively (186).

### Acetylation and TCA Cycle Enzymes

Acetyl-CoA is the final common product in the oxidative metabolism of various fuels, and is a substrate for the TCA cycle. While acetylation of all TCA cycle enzymes have been reported in the liver (20), 6 of the 8 enzymes were found to be acetylated in the heart (151). However, examination of the effect of acetylation on the TCA cycle has produced mixed results. Increased acetylation of malate dehydrogenase (MDH) in Chang liver cells enhances its enzyme activity. When cells were treated with deacetylase inhibitors, trichostatin A (TSA) and NAM, MDH acetylation doubled the endogenous MDH activity, while *in vitro* deacetylation of purified MDH decreased its activity (20). Similarly, significant acetylation-dependent activation of aconitase was found in both isolated heart mitochondria subjected to *in vitro* chemical acetylation, and in hearts of HFD fed obese mice (152). Increased aconitase acetylation at multiple sites were found, with acetylation at K144 identified as a responsible site for structural change at the active site that was reversed by increasing SIRT3 overexpression (152).

Although acetylation at multiple sites was detected on the TCA cycle enzyme isocitrate dehydrogenase (IDH), no significant effect of this acetylation on enzyme activity was found (151). In contrast, others have reported a significant loss of function of IDH when acetylated at lysine 413, which is fully restored by SIRT3 mediated deacetylation (187). Additionally, increased acetylation of succinate dehydrogenase (SDH), which functions both in the TCA cycle and electron transport chain, is associated with a decrease in activity in human and mice heart failure (81). Further investigation on its acetylation sites revealed that lysine 179 of SDH as an important site for acetylation that regulates enzyme activity by interfering in FAD^+^ binding to the enzyme. However, despite widespread acetylation of its enzymes, the overall TCA cycle activity appears to be unaffected by excessive acetylation (83).

Succinylation and malonylation of TCA cycle enzymes have also been reported (25, 26, 142). However, the impact of this succinylation and malonylaton has been assessed on only a few enzymes. SIRT5 mediated desuccinylation activates IDH (188). Paradoxically, SDH desuccinylation has been suggested to inhibit its activity in MEFs, while SIRT5 deletion leads to an increase in SDH activity (25).

### Acetylation of Proteins in Electron Transport Chain and Oxidative Phosphorylation

Lysine acetylation of mitochondrial respiratory complex enzymes, NADH dehydrogenase 1, ubiquinol cytochrome c reductase core protein 1, and ATP synthase mitochondrial F1 complex assembly factor 1 is increased in mice hearts that lack SIRT3. In these hearts, functional studies demonstrated inhibition of Complex I activity (24). In another study, a similar effect was observed in neonatal rat cardiomyocytes, as well as in H9c2 cells treated with high glucose, oleate, and palmitate (153). Treatment with HDACs and sirtuin inhibitors, TSA and NAM, respectively, further increase the levels of acetylated proteins in mitochondrial complexes I, III, and V, with a concomitant decrease in ATP production. However, treatment with exogenous H_2_S elevates the NAD^+^/NADH ratio and the activity of SIRT3, both of which are decreased in the presence of high glucose and fatty acid as well as in diabetes (153). Similarly, a decrease in the activity of complex V in association with increased acetylation was shown by Kerner et al. (151).

### Nuclear Acetylation Control of Mitochondrial Metabolism

Acetylation can also affect transcription factors that regulate energy metabolism. Peroxisome proliferators-activated receptors (PPARs) are a family of nuclear receptors that have a critical role in regulating the expression of proteins involved in fatty acid metabolism. The PPAR transcription factors, comprised of PPARα, PPARδ, and PPARγ, form a complex with retinoid X receptor (RXR) and bind the peroxisome proliferator response element (PPRE) in the promoter region of target genes, thereby initiating their transcription (189, 190). Similarly, estrogen receptor-related receptors (ERRs) including ERR α, β, and γ affect the expression of enzymes in the glucose and fatty acid metabolic pathways after binding with ERR responsive elements (191). Peroxisome proliferator-activated receptor-gamma coactivator-1 alpha (PGC-1α) is an inducible cofactor of both PPARs and ERRs (192). Activation of PGC-1α together with PPAR and ERR promotes fatty acid utilization while suppressing glucose metabolism. All of these transcription factors have been shown to be subject to acetylation/acylation.

SIRT1 interaction with PPARα positively affects PPARα activity. While SIRT1 deficiency impairs PPARα signaling and decreases fatty acid ß-oxidation, while its overexpression upregulates PPARα targets (193). On the contrary, SIRT4 decreases PPARα activity and consequently the expression of PPARα target genes (194). Acetylation of PPARγ at different lysine residues has been shown in various tissues (195, 196). Repression of PPARγ by SIRT1 is seen in 3T3-L1 adipocytes (197). Activation of PPARγ has been seen with acetylation and its suppression by histone deacetylase 3 (HDAC3) (198). In contrast, PGC-1α is deacetylated and activated by SIRT1 (199–201). ERRα is another transcription regulator that is modified by acetylation with suppressed sensitivity after acetylation (202).

Altered energy metabolism may also influence the expression pattern of metabolic genes through chromatin modification by post-translational histone acetylation. Accordingly, increased glucose supply leads to increased histone acetylation with a corresponding activation of glucose metabolism genes *in vitro* (203). Similarly, upregulation of several lipid metabolism-related genes is observed in response to fatty acid-derived acetyl-CoA-induced histone hyperacetylation (204). While these data support substrate-dependent induction of specific metabolic genes, it unclear how cells respond differently to the acetyl-CoA derived from either glucose and fatty acid ß-oxidation. Additionally, the contribution of histone acetylation modifications as a result of metabolite imbalances has not been determined in the transcription dysregulation seen in pathological states.

## Protein Acetylation in Heart Failure

**Figure 3:**
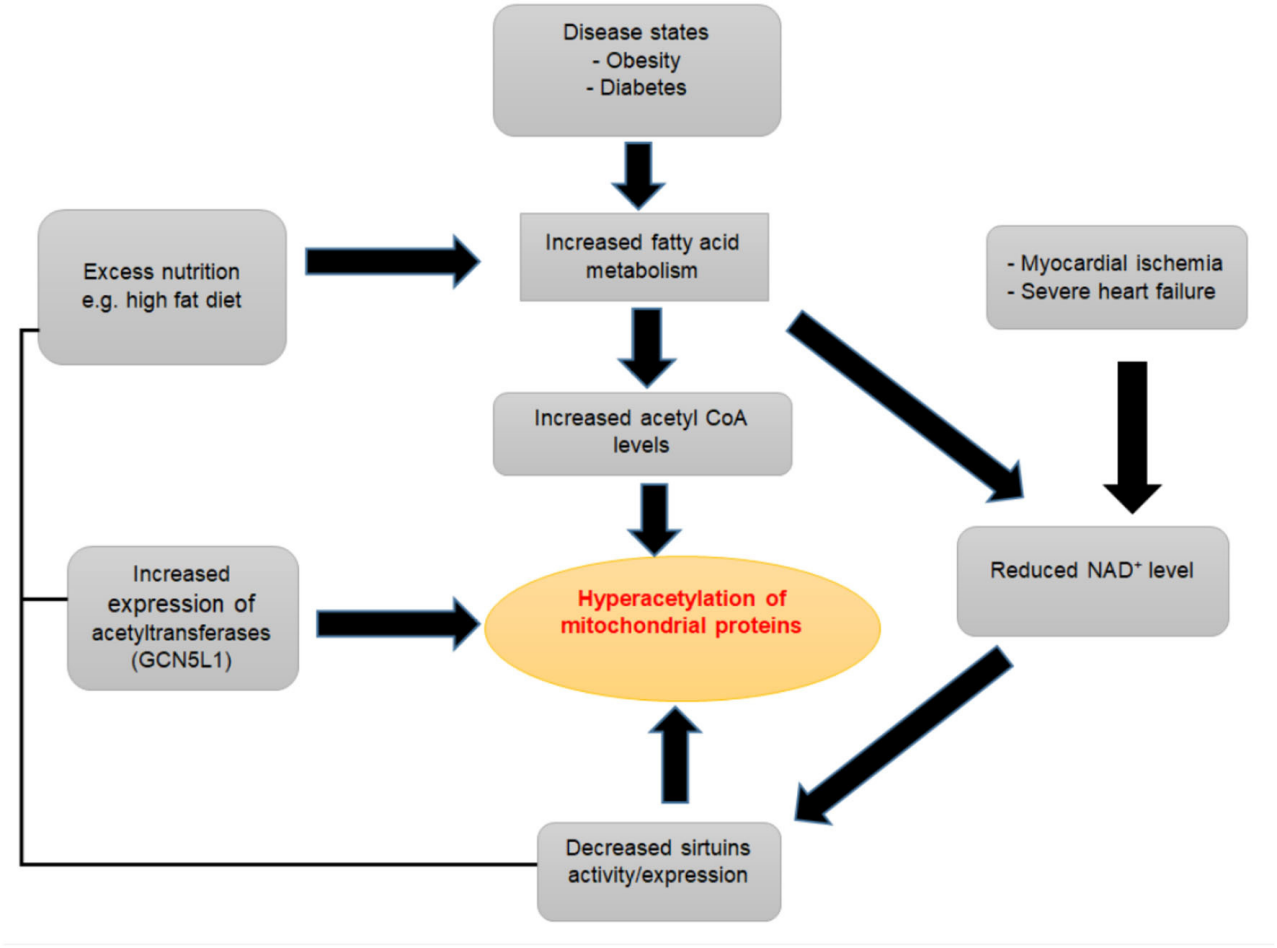
Contributing factors to mitochondrial protein hyperacetylation.

Studies in both animal models and humans have shown increases in acetylation of mitochondrial proteins in the failing heart compared to healthy control hearts (Figure 3) (80, 81). Davidson et al. compared the acetyl proteomics profile between dual KO mice for SIRT3 and carnitine acetyltransferase (which causes extreme mitochondrial acetylation) and TAC induced heart failure mice (159). These authors found an approximately 86% overlap in acetylated peptides between the double KO mice and the experimental heart failure mice. Furthermore, in rat models of hypertensive heart failure, the Dahl salt-sensitive (SS) and spontaneously hypertensive heart failure-prone (SHHF) rats, a large number of proteins were found exclusively hyperacetylated in the failing hearts compared to control hearts (140). Increased acetylation was accompanied by a reduced level of SIRT3 in these pressure-overload-induced failing hearts (140). Similarly, in failing hearts of obese patients, Castillo et al. found a 46% decline in SIRT3 expression and increased acetylation profiles in heart failure patients with a BMI >30, as well as in obese rat hearts (205). Increased cardiac acetylation was also observed in obesity-related left ventricular remodeling and cardiac fibrosis (206). A dramatic increase in protein lysine acetylation was also seen in the heart and mitochondria from diabetic mice, along with decreased deacetylation reactions (146). Several other studies have also revealed the abundance of hyperacetylation of different proteins in various forms of heart failure (207–209). Although these data have consistently shown the increased protein acetylation in heart failure settings, the specific impact, regulations, and the mechanisms of how these changes are linked to heart failure remain incompletely understood. Moreover, the mass spectrometry-based acetylome proteomics used in these studies have inherent limitations to measure acetylation changes at the protein level and individual acetylation sites within each protein, which would be necessary to understand the biological significance of acetylation (210–213).

### Acetylation and Shifts in Myocardial Energy Metabolism During Heart Failure

Numerous studies have demonstrated altered substrate preferences and metabolism in the failing heart [see (28) for review]. However, the actual direction of these shifts in energy substrate utilization remains controversial. While the shift toward increased oxidation of fatty acid is widely observed in ischemic and diabetic heart failure, other studies have suggested a shift in myocardial metabolism away from fatty ß-oxidation and its association with the progression to ventricular dysfunction in hypertrophic hearts (214). According to the principles outlined by Randle (215), glucose and fatty acid metabolism are regulated reciprocally. As highlighted in the preceding sections, lysine acetylation affects the main enzymes of both fatty acid and glucose oxidation inversely in the heart. In fact, various animal studies, as described below, have demonstrated that acetylation may be sufficient to cause shifts in myocardial substrate preference in the presence of pathological stressors.

In type 1 diabetic mice hearts, increased acetylation induces mitochondria metabolic inflexibility accompanied by decreased activities of PDH and complex II enzyme activities (85). A dramatic decrease in mitochondrial respiration in the presence of non-fatty acid substrates was observed in contrast to minimal inhibition in palmitoylcarnitine-supported respiration (85). Similarly, other studies have shown a switching in cardiac energy metabolic substrate preference toward fatty utilization by lysine hyperacetylation in type 2 diabetes mice (216). In *db/db* mice, and in cardiomyocytes in culture, increased acetylation of enzymes involved in mitochondrial fatty acid ß-oxidation and glucose oxidation were found, with a concomitant decrease in the expression and activity of SIRT3. While LCAD acetylation is accompanied by a significant upregulation in its activity, the hyperacetylation of PDH is associated with a decrease in its activity. In support of this, decreasing acetylation by increasing the expression and activities of SIRT3 (through exogenous hydrogen sulfide administration) switches cardiac substrate utilization from fatty acid ß-oxidation to glucose oxidation in diabetic mice hearts (216).

Additionally, Romanick et al. observed marked changes in lysine acetylation in cardiac tissues with obesity (206). Of those significantly impacted by increased acetylation due to obesity were very long-chain specific acyl-CoA dehydrogenase, aconitate hydratase 2, and dihydrolipoyl dehydrogenase. Interestingly, increased transcriptional activation of KLF15 and PPARα, with increased expression of downstream target genes and their interaction with these significantly acetylated proteins, was observed in diet-induced obesity. In addition, the authors of this study found enhanced expression of PDK4 and MCD in the heart (206). PDK4 is known to inhibit glucose oxidation by inhibiting pyruvate dehydrogenase, while malonyl CoA decarboxylase promotes fatty acid ß-oxidation via activation of carnitine palmitoyltransferase 1 (CPT1). Together this suggests that shifting toward fatty acid ß-oxidation, at the expense of glucose oxidation, occurs in the presence of hyperacetylation (206). We and others have also observed that hyperacetylation on PDH, LCAD, and β-HAD, accompanied by a decrease in glucose oxidation and an increase in fatty acid oxidation, is seen in the heart in response to HFD feeding (28, 53). Likewise, as discussed in the preceding sections, in skeletal muscle a switch in substrate utilization from glucose oxidation toward fatty acid utilization is also induced by SIRT3 KO, as a result of PDH inhibition by hyperacetylation of its E1α subunit (156). Thus, acetylation could contribute to the metabolic inflexibility seen in heart failure by regulating metabolic enzyme activities differently.

Myocardial I/R is another pathology where shifts in energy substrate utilization are implicated in heart injury. Increased fatty acid ß-oxidation rates following ischemia result in suppression of glucose oxidation and a subsequent uncoupling of glucose oxidation from glycolysis, which contributes to ischemic damage (217, 218). As discussed in the preceding section, the increased acetylation of proteins following enhanced fatty acid ß-oxidation and increased acetyl-CoA generation may contribute to the metabolic phenotype observed in I/R. In addition, as NAD^+^ availability is a critical determinant for Sirtuin activity, ischemia-induced decreases in the NAD^+^/NADH redox couple during I/R may inactivate SIRT3 and lead to the hyperacetylation of mitochondrial proteins (80). Furthermore, the mRNA and protein levels of NAMPT, the rate-limiting enzyme that converts NAM to NMN in the NAD^+^ salvage synthesis pathway is downregulated in the heart during I/R injury, further reducing NAD^+^ levels and thus Sirtuin activity (219). Several studies have demonstrated the cardioprotective role of NAD^+^ in I/R injury either by exogenous NAD^+^ supplementation or enzymatic manipulation (80, 125, 219, 220). However, this mechanism has not yet been investigated in relation to changes in metabolic enzymes activity or metabolic alterations as a result of acetylation suppression by NAD^+^ boosting. There is little data that support the idea that this effect may be related to increased glucose oxidation rates. Accordingly, in rats subjected to I/R after prolonged caloric restriction, Shinmura et al. showed a decreased level of acetylated mitochondrial proteins associated with enhanced Sirtuin activity and attenuated myocardial oxidative damage. Interestingly, caloric restriction increases respiratory control index and oxygen consumption in the presence of pyruvate/malate substrates in mitochondria isolated from I/R hearts (221). However, the hearts in this study were reperfused for only 3–5 min after ischemia and the proteomic analysis was not robust enough to analyze the acetylome changes in metabolic enzymes.

### Contribution of Hyperacetylation to Cardiac Dysfunction

As discussed above, hyperacetylation of myocardial proteins is common in heart failure. In addition to its regulatory role in myocardial energy metabolism, several studies have also analyzed the impact of hyperacetylation of myocardial proteins on heart failure development and progression. While the majority of the studies suggested a link between hyperacetylation and worsening of heart failure (80, 81, 105, 146, 206, 222, 223), others found no association between myocardial dysfunction and hyperacetylation (159, 224). The cause for the discrepancies in these studies awaits further studies.

Hypertrophy is one of the pathological processes where increased acetylation is implicated in disease progression. In response to various hypertrophic stimuli, SIRT3-deficient mice appear to be more sensitive to injuries and manifested various abnormalities compared to their wild-type counterparts (105). On the other hand, SIRT3 transgenic mice are protected from hypertrophic injuries (105). Activation of forkhead box O3a-dependent (Foxo3a) and manganese superoxide dismutase and catalase are also seen in response to SIRT3 activation. Activation of SIRT3 has been also implicated in lessening the severity of cardiac hypertrophy by blocking interstitial fibrosis, as well as fibroblast proliferation and differentiation (222). Furthermore, in SIRT5 KO mice subjected to TAC, Herschberger et al. observed a reduced survival of SIRT5 KO mice compared with wild-type mice (225). The increased pathological hypertrophy and mortality in these mice is associated with several biochemical abnormalities including reduced fatty acid ß-oxidation and glucose oxidation, suggesting that SIRT5-mediated desuccinylation plays an important role in regulating cardiac metabolism during stress.

Changes in protein acetylation have been also shown in myocardial I/R injury in association with decreased SIRT3 protein levels (158, 223, 226). In support of this, increased mitochondrial protein acetylation in SIRT3 KO mice is associated with increased sensitivity to injury, as shown by larger infarct size, less functional recovery, and low O_2_ consumption rates (223). In contrast to these findings, the study by Koentges et al. found no additional susceptibility to I/R-specific injury in SIRT3 KO mice that underwent permanent ligation of the left anterior descending coronary artery (LAD) (125). On the other hand, Boylston et al. found an increase in infarct size and impaired recovery during I/R in SIRT5 KO hearts compared to WT littermates (97). This injury was decreased by pretreatment with dimethyl malonate, a competitive inhibitor of SDH, suggesting that the enhanced activity of SDH by hypersuccinylation is an important cause for increased ischemic injury (which inconsistent with other reports) (25). Similarly, using exogenous NAD administration, Liu et al. demonstrated that SIRT5-mediated SDH desuccinylation decreased the activity of SDH, which attenuated the succinate accumulation during I/R and alleviated reactive oxygen species generation (227). In a separate study, accumulation of succinate during ischemia and its rapid oxidation by SDH during reperfusion can drive extensive ROS generation in a murine I/R injury model (228).

Though complete data are unavailable on the acetylation status of metabolic enzymes and its impact specifically in I/R, several studies have investigated this modification in other proteins or pathways. The permeability transition pore (PTP) is an important inner membrane channel that has a role in I/R injury (229). Permeability transition pore opening is facilitated by the translocation of cyclophilin D (CyPD) from matrix protein to the inner mitochondrial membrane (230). The study by Bochaton et al. in SIRT3 KO mice demonstrated that increased acetylation of CyPD following myocardial I/R facilitates PTP opening and subsequent cell death, which was prevented by attenuation of CyPD acetylation at reperfusion (231). A similar result was found in mice subjected to renal I/R where dexmedetomidine induced SIRT3 overexpression and significantly reduced I/R related mitochondrial damage by decreasing cyclophilin D acetylation (232).

## Summary

Although excessive mitochondrial metabolic enzyme acetylation occurs in heart failure, its contribution to cardiac metabolic alterations remains incompletely defined. However, accumulating evidence supports the hypothesis that acetylation alters energy substrate utilization in heart failure by activating fatty acid ß-oxidation and inhibiting glucose oxidation. While the inhibitory effect of acetylation on glucose oxidation is widely accepted, there are still disagreements as to the relationship between acetylation and fatty acid ß-oxidation. While most of the studies done in the heart and skeletal muscle tissues illustrate a positive association between acetylation and fatty acid ß-oxidation, others studies suggest a suppressive effect of acetylation on fatty acid ß-oxidation in the liver. Tissue or site-specific variation in acetylation of these enzymes, as well differences in underlying pathologies, could contribute to such discrepant data. Available data also suggests that acetylation does not always has an inhibitory effect on metabolic enzymes.

## Author Contributions

All authors listed have made a substantial, direct and intellectual contribution to the work, and approved it for publication.

## Conflict of Interest

The authors declare that the research was conducted in the absence of any commercial or financial relationships that could be construed as a potential conflict of interest.

## Publisher's Note

All claims expressed in this article are solely those of the authors and do not necessarily represent those of their affiliated organizations, or those of the publisher, the editors and the reviewers. Any product that may be evaluated in this article, or claim that may be made by its manufacturer, is not guaranteed or endorsed by the publisher.
